# The Mechanisms of the Potential Probiotic *Lactiplantibacillus plantarum* against Cardiovascular Disease and the Recent Developments in its Fermented Foods

**DOI:** 10.3390/foods11172549

**Published:** 2022-08-23

**Authors:** Zhe Wang, Juanjuan Wu, Zichen Tian, Yue Si, Hao Chen, Jing Gan

**Affiliations:** 1 Marine College, Shandong University, NO. 180 Wenhua West Road, Gao Strict, Weihai 264209, China; 2 College of Life Science, Yantai University, Yantai 264000, China

**Keywords:** cardiovascular disease, *Lactiplantibacillus plantarum*, fermentation, probiotic functional foods, probiotics, nutriceuticals

## Abstract

Cardiovascular disease (CVD) has become the leading cause of death worldwide. Many recent studies have pointed out that *Lactiplantibacillus plantarum* (*Lb. plantarum)* has great potential in reducing the risk of CVD. *Lb. plantarum* is a kind of lactic acid bacteria (LAB) widely distributed in fermented food and the human intestinal tract, some strains of which have important effects on human health and the potential to be developed into probiotics. In this review, we summarize the mechanism of potential probiotic strains of *Lb. plantarum* against CVD. It could regulate the body’s metabolism at the molecular, cellular, and population levels, thereby lowering blood glucose and blood lipids, regulating blood pressure, and ultimately reducing the incidence of CVD. Furthermore, since *Lb. plantarum* is widely utilized in food industry, we highlight some of the most important new developments in fermented food for combating CVD; providing an insight into these fermented foods can assist scientists in improving the quality of these foods as well as alleviating patients’ CVD symptoms. We hope that in the future functional foods fermented by *Lb. plantarum* can be developed and incorporated into the daily diet to assist medication in alleviating CVD to some extent, and maintaining good health.

## 1. Introduction

Cardiovascular disease (CVD) has become much more prevalent and complicated as our modern society develops. CVD is one of the largest contributors to the burden of chronic disease, with an estimated 17.9 million people dying from it in 2019 globally [1]. It is also reported that CVD was the leading cause of death in 2018 in China, accounting for 46.66% and 43.81% of all deaths in rural and urban areas, respectively [2]. Also, despite the lowered incidence of CVD in adults aged >50 years, the incidence of CVD has either been steady or increasing among younger adults (aged 18–50 years) in Western countries over the past two decades [3]. Thus, not only the elderly but also young people should attach importance to cardiovascular health.

**Figure 1:**
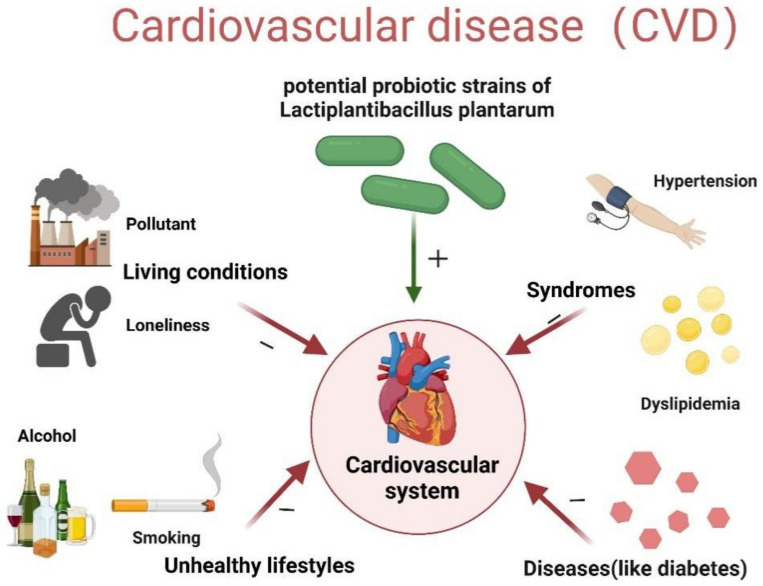
**Impacts of different factors on cardiovascular health.** There are mainly many causes that will lead to cardiovascular disease (CVD) and potential probiotic strains of *Lactiplantibacillus plantarum* (*Lb. plantarum*), one kind of lactic acid bacteria (LAB), may have the ability to protect the cardiovascular system. (“−“means that this factor is harmful to the health of the cardiovascular system, and “+” means that this factor promotes the health of the cardiovascular system) (Created with Biorender.com).

The factors that influence our cardiovascular health are complicated and multiple, as illustrated in Figure 1. Diseases, syndromes, personal lifestyles, and living conditions will all have an impact on our cardiovascular health. CVD is the most common complication in certain diseases, such as diabetes mellitus, and has now become the leading cause of death in people with diabetes [4]. In the meantime, according to a review study in 2022 [5], syndromes like hypertension and dyslipidemia are important CVD risk factors for people. Moreover, adhering to a healthy lifestyle, including regular fruit intake, less alcohol consumption, and non-smoking, could statistically significantly reduce the overall incidence of CVD by 66% [6]. Different kinds of environmental stressors, such as discrimination, social isolation, low socioeconomic status, can alter an individual’s biology through many kinds of pathways, such as the neuro-hematopoietic axis and epigenetic modification. This will lead to chronic inflammation and, finally, result in the development and progression of CVD [7]. Scientists also find that people who are continuously exposed to hazardous waste will have a tendency to get high blood pressure [8]. As a result, finding a way to reduce the risk of CVD is an important health-related topic in modern people’s lives nowadays. 

In these circumstances, besides the urgent need for new effective medicines, the development of probiotics and probiotic foods has also become a potential way to protect people against CVD. Probiotics are defined as “live microorganisms which when administered in adequate amounts confer a health benefit on the host” [9]. The *Lactobacillus* spp. are widely considered to be probiotics because of their generally probiotic properties [10]. *Lactiplantibacillus plantarum* (*Lb. plantarum*), a species of the *Lactobacillus* spp., is also considered to be a potentially probiotic strain, with some *Lb. plantarum* strains able to help maintain the balance of blood glucose, lipids, and blood pressure, and to regulate metabolic disorders [11], thus having anti-cardiovascular potential. The entry of probiotics into the human gastrointestinal tract requires the use of carriers, usually including capsules and food products, with the latter offering better acceptance and marketing than the encapsulation technology. Fermentation is an effective method to produce probiotic foods. *Lb. plantarum* with probiotic potential can be used as a fermentation strain for a variety of foods, such as dairy products, meat products, vegetables and fruits, and cereals. Several studies have shown that foods fermented with *Lb. plantarum* have higher health benefits, such as better antioxidant capacity and higher active substance content [12,13]. In addition, scientists have also found that *Lb. plantarum*-fermented foods may also be endowed with certain probiotic effects, and may be developed into probiotic functional foods that can be useful for human health through daily dietary supplementation.

This review shows the probiotic potential of *Lb. plantarum* and analyzes the possible mechanisms by which *Lb. plantarum* can prevent or help improve the condition of CVD. In addition, this review focuses specifically on the great potential of *Lb. plantarum*-fermented foods to be developed as probiotic functional foods, providing an insight into how these fermented foods could assist scientists in improving their quality and ultimately alleviating patients’ CVD symptoms.

## 2. Probiotic Potential of *Lb. plantarum* and Foods Fermented with It

*Lb. plantarum* belongs to the genus *Lactobacillus*, which is widely considered to be a probiotic, and may have probiotic potential from species analysis. However, a prerequisite for probiotics to function in the host’s gastrointestinal tract is a good tolerance to unfavorable factors such as gastric acid, bile salts, and degrading enzymes, as well as the ability to complete colonization in the gastrointestinal tract [14]. Therefore, *Lb. plantarum* must have a high gastrointestinal survival rate to meet the basic requirements for development as a probiotic. Kriti Ghatani and Buddhiman Tamang [15] isolated a strain of *Lb. plantarum* from fermented yak milk products, and then added cholesterol and bile salts to MRS broth to determine the tolerance of the selected strain. Combined with acid resistance, bile salt hydrolase activity and cell surface hydrophobicity, they confirmed that this strain of *Lb. plantarum* was tolerant to the corrosive and toxic effects of gastric acid and bile in humans, and can adhere to host cells to complete colonization. Wang, G.Q. and Chen, Y. et al. [16] recorded the images of mouse intestines at different times after oral administration of *Lb. plantarum*, confirming that *Lb. plantarum AR17-1* can colonize and play a role in the intestinal tract, implying that *Lb. plantarum* has probiotic potential.

As a carrier for *Lb. plantarum* to enter the human body, *Lb. plantarum*-fermented foods with high live counts have the potential to be developed into probiotic foods that can play a healthy role in the human body. However, the ability of these fermented foods to function as probiotic foods also depends on the gastrointestinal survival of the probiotic bacteria in the food. Different food matrices also have significant impacts on the gastrointestinal survival rate of *Lb. plantarum*. The most widely used matrices are dairy products including yogurt, cheese, etc. The higher fat and whey protein content in dairy products result in their better buffering capacity against gastric acid, which can improve the gastrointestinal survival rate of probiotics [17,18]. In addition, other fermented foods, such as fruit and vegetable juices, oats and cereals, and meat products are also commonly used as food matrices to create fermented foods that bring probiotics into the human gastrointestinal tract. The fat in meat products protects probiotics from low pH and bile salts [18], the higher sugar content in cereals allows probiotics to better tolerate intestinal conditions [19], and the relatively short digestion time of fruit and vegetable juices can greatly reduce the adverse effects of the gastric environment on probiotics [20,21]. Current *Lb. plantarum*-fermented foods that may have probiotic potential include dairy products, meat products, soy products, fruits and vegetables, and cereals, where the gastrointestinal survival of *Lb. plantarum.* is high. However, studies on the effect of food matrices on the gastrointestinal survival rate of probiotics are still relatively few, and it is hoped that more research will focus on this in the future.

## 3. Possible Mechanism of *Lb. plantarum* against CVD

### 3.1. Mechanisms of Antioxidant Abilities by Lb. plantarum

Free radical generation has been linked in studies to the development of CVD. It has been demonstrated that several strains of *Lb. plantarum* achieve antioxidant capacity by scavenging free radicals. Therefore, we speculate that the *Lb. plantarum* strains with antioxidant capacity may have the potential to mitigate CVD [22].

The antioxidant mechanisms of *Lb. plantarum* are complex and diverse. For example, *Lb. plantarum JM113* can increase the mRNA levels of Nrf2 (a protein that regulates endogenous antioxidant systems) and its corresponding downstream HO-1 gene (HO-1, an important antioxidant enzyme that mainly catalyzes the decomposition and metabolism of heme to ferrous, carbon monoxide, and bile green) to achieve antioxidation [23]. *Lb. plantarum KCCP11226* can produce C-30 carotenoid 4,4’-diaponeurosporene, which has antioxidant capacity [24,25,26]. *Lb. plantarum DM5* has been reported to directly scavenge superoxide anion radical, hydroxyl radical, and DPPH radical [27,28]. Further mechanisms for the antioxidant activity of *Lb. plantarum* are shown in Table 1.

On the other hand, a single *Lb. plantarum* may have several different antioxidant mechanisms. For example, *Lb. plantarum NJAU-01* can not only alleviate and protect the body from oxidative stress by regulating the protein expression in the metabolic pathways, but also regulate the composition of the intestinal flora. Additionally, its extracts also show powerful antioxidant capacity [29,30].

In addition, the antioxidant capacity and antioxidant mechanisms of *Lb. plantarum* may be affected by several environmental conditions and interactions with other strains. For example, hydrogen peroxide (H_2_O_2_) is one of the antagonism factors to regulate the gut microbiota composition, and after being stimulated by H_2_O_2_, the redox balance in the *Lb. plantarum KM1* is destroyed, resulting in the down-regulation of the expression of α-glycerophosphate oxidase and pyruvate oxidase, and the reduction of H_2_O_2_ production in the bacteria, thus achieving an antioxidant effect [31].

The antioxidant function of *Lb. plantarum* has been proved in animal experiments, with several strains of *Lb. plantarum* exerting stable antioxidant effects in mice [32,33]. For example, in the experiment of Yan, L. et al. [34], a certain dose of *Lb. plantarum FEED8* solution and the corresponding dose of skimmed milk were intragastrically administered to mice in the two groups. The antioxidant effect was evaluated by detecting the superoxide dismutase (SOD) activity, glutathione peroxidase (GSH-Px) activity, total antioxidant capacity (T-AOC) content, and malonaldehyde (MDA) content in the serum, brain, and liver of mice. The results showed that compared with the control group, the SOD activity, GSH-Px activity, and T-AOC content in serum, brain, and liver of mice in the medium and high dose *Lb. plantarum FEED8* groups were significantly increased, and the MDA content was significantly decreased. This difference grew as the concentration of *Lb. plantarum FEED8* increased. It can be concluded that *Lb. plantarum FEED8* has good antioxidant effect on mice. This gives some indication of whether *Lb. plantarum* can exert antioxidant effects on humans.

**Table 1:** Possible mechanisms by which *Lb. plantarum* protects cells from the attack of free radicals.

Name of *Lb. plantarum*	Resource	In Vivo Studies(a) or In Vitro Studies(b)	Antioxidant Mechanism	Reference
*Lb. plantarum CCFM10*	Different storage centers (edible fungi strains)	a	Increase levels of GSH, CAT, SOD, and TOC in serum of mice with oxidative damage	[35]
*Lb. plantarum CCFM242*	Different storage centers (edible fungi strains)	a	Improve total antioxidant capacity of liver	[35]
*Lb. plantarum RS15-3*	Different storage centers (edible fungi strains)	a	Increase levels of GSH, CAT, SOD, and TOC in serum of mice with oxidative damage	[35]
*Lb. plantarum FEED8*	Intestinal tract of longevity elderly in Bama, Guangxi	a	Increase levels of glutathione (GSH) and other indicators	[34]
*Lb. plantarum NCFM, ATCC 14917 and NDC 75017*		a	Increase SOD activity, GSH-Px activity, and T-AOC content in serum, brain, and liver, decreasing MDA content.	[26]
*Lb. plantarum C88*	Traditional fermented milk tofu in Inner Mongolia	a	Regulation of intracellular antioxidant enzyme activity in oxidative damaged Caco-2 cells	[36]
*Lb. plantarum Y-20*	Chopped pepper by natural fermentation	a	Protect the Caco-2 cells against H_2_O_2_ and induce oxidative stress by renewing the enzymatic and non-enzymatic antioxidant defense system.	[37]
*Lb. plantarum JMCC0017*	Xinjiang traditional fermented dairy products	b	Metabolites have significant antioxidant activity	[38]
*Lb. plantarum DM5*	Fermented beverage Marcha of Sikkim	a	Scavenge free radicals and superoxide anion	[27,28]
*Lb. plantarum KM1*	Natural fermentation products	a	Scavenge hydroxyl radical, superoxide anion radical, and DPPH radical	[31]
*Lb. plantarum AR501*		a	Scavenge hydroxyl radical, superoxide anion radical, and DPPH	[39]
*Lb. plantarum JM113*	healthy intestinal contents of Tibetan chicken	a	Change the expression levels of Phosphoglycerin kinase, α-glycerophosphate oxidase, pyruvate oxidase, and NADH peroxidase to alleviate oxidative stress.	[23]
*Lb. plantarum NC8*		a	Up-regulation of antioxidant gene expression	[40]
*Lb. plantarum SM4.*	kimchi and fermented with white Taraxacum coreanum	a	Increase the mRNA levels of Nrf2 and its corresponding downstream HO-1 gene	[41]
*Lb. plantarum KCCP11226*		a	Regulation of Bcl-2 family members and activation of Bcl-2/Bax signaling pathway	[24,25]
*Lb. plantarum ZLP001*		b	Produce white T. coreanum fermented product which shows higher bioactive properties of oxidation resistance	[42]
*Lb. plantarum KLDS1.0202*	Cheddar cheese	b	Produce C-30 carotenoid 4,4′-diaponeurosporene.	[43,44]
*Lb. plantarum* isolated from traditional sourdough	Traditional Sourdough	a	Supplementation of Lb. plantarum ZLP001 increases the concentration of superoxide dismutase (p < 0.05), glutathione peroxidase (p < 0.01), and catalase in serum (p < 0.10), while decreasing the concentration of malondialdehyde (p < 0.05).	[45]
*Lb. plantarum NJAU-01*	jinhua ham		The strain not only itself has a certain antioxidant activity, but also promotes the decomposition of protein of Cheddar cheese	[29,30]

### 3.2. Mechanisms of Blood Pressure Lowering by Lb. plantarum

Hypertension, defined as systolic blood pressure (BP) ≥130 mmHg and/or diastolic BP ≥80 mm Hg [46], has become much more prevalent in recent years. Due to increases in unhealthy lifestyles, including high sodium diets, low potassium intake, alcohol consumption, overweight condition, and lack of exercise, hypertension is now becoming a leading cause of CVD [47]. According to the statistics generated by M. H. Forouzanfar, et al. [48], hypertension led to the deaths of 7.8 million people (14.0% of all deaths) in 2015.

Angiotensin-converting enzyme (ACE) contributes significantly to regulating blood pressure. According to the research conducted by Eriksson in 2002 [49], with the elevation of ACE activity, BP will rise because ACEs can promote the production of angiotensin, which can cause vascular contraction. Recently, some studies have shown that foods fermented by *Lb. plantarum* could assist in lowering patients’ BP. In a systematic review and meta-analysis, the authors found that when hypertensive patients ingested *Lb. plantarum* supplementation, their diastolic BP reduced significantly compared with normal people [50]. According to Chen et al. [51], goat milk fermented by *L. plantarum 69* could inhibit ACE activity, with the calculated inhibition rate reaching 88.91%. Some other findings regarding the inhibitory capacity of these functional foods on ACE activity are presented in Table 2.

**Figure 2:**
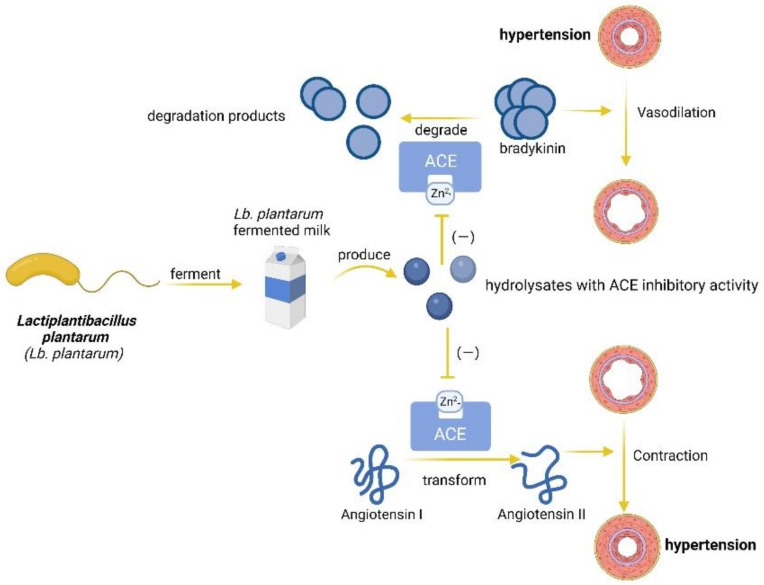
**The blood pressure-lowering mechanism of *Lactiplantibacillus plantarum* fermented food.***Lactiplantibacillus plantarum (Lb. plantarum)* fermented foods can produce angiotensin-converting enzyme (ACE) inhibitors, which can prevent bradykinin from degrading and angiotensin I from transforming, thus relieving hypertension symptoms. (“−“ means inhibition) (Created with Biorender.com).

The specific proteolytic system of potentially probiotic strain *Lb. plantarum* might be the main reason for its inhibition of ACE activity [52]. According to Fabiola Sanchez-Lopez et al. [53], the product obtained after 8 h of hydrolysis of amaranth proteins by *Lb. plantarum* exhibited favorable ACE-inhibitory (ACEI) activity. These hydrolysates, whose chemical essence is mainly peptides, could chelate Zn^2+^ to eventually reduce the activity of ACE. Moreover, Yi-Yen Liu et al. [54] reported that one kind of bioactive ACEI peptide extracted from soy milk could promote nitric oxide production after being fermented by *Lb. plantarum TWK10*. With the increase of nitric oxide level in cells, blood vessels can be relaxed, contributing to further improvement in hypertension. Nahariah, N. et al. [55] reported that egg albumen fermented by *Lb. plantarum FNCC 0027* for 18 h was rich in ACEI peptides. However, the longer fermentation periods (24, 30, and 36 h) reduced the ACEI peptides’ activities in fermented egg albumen. In addition, Shu, Guowei [56] reported that the optimal temperature for one kind of milk to ferment and induce ACEI peptide activity was 35 °C. In the meantime, digestive enzymes also have a great impact on these functional foods. If digestive enzymes, such as pepsin and chymotrypsin, can destroy these ACEI peptides, these functional foods will scarcely have any benefits. According to Yanan Xia et al. [57], ACEI peptides isolated from whey proteins of milk fermented with *Lb. plantarum QS670* exhibited stability toward chymotrypsin, pepsin, and trypsin, which meant that the fermented milk can be used as a potential functional food to help relieve the symptoms of hypertension. The mechanism by which *Lb. plantarum* lowers ACE activity and reduces blood pressure is illustrated in Figure 2. However, the impact of digestive enzymes is sometimes overlooked by researchers in in vitro tests, so some results of these kinds of experiments will be further illustrated through in vivo tests.

In the meantime, γ-aminobutyric acid (GABA), a non-protein amino acid, can act as a hypotensive agent. Some studies have indicated that GABA can reduce high blood pressure in both animals and humans [58]. Since the GAD enzyme (a key enzyme to synthesize GABA) is highly active in the cells of LAB, they have high GABA production. Mohsen Zareian et al. [59] reported that using *Lb. plantarum MNZ* to ferment a bowl of wheat-based rice (dosa) for 120 h could enhance the GABA content (about 143 mg/kg) of the dosa. GABA could also be found when different types of *Lb. plantarum* are used to ferment milk [60] and lentils [61].

Apart from ACEI peptides and GABA, certain kinds of phenolic acids can also contribute to lowering blood pressure. According to Irini Lazou Ahrén et al. [62], blueberries fermented by *Lb. plantarum DSM 15313* can produce three kinds of phenolic acids (hydroxyphenyllactic acid, 3,4-dixydroxyphenyl-propionic acid, and phenyllactic acid). They separated rats into six groups. Three groups had normal blood pressure and were then fed with standard chow, fermented blueberry products with a low concentration of the phenolic acids (product A), and products with a high level of the same (product B). Another three groups were induced hypertension and then fed with the same three kinds of foods. The authors found that after feeding normal rats with product A for four weeks, their blood pressure dropped more obviously than the other two groups. Nevertheless, product B can significantly reduce hypertensive rats’ blood pressure compared with product A. The conclusion was that phenolic acids have the ability to reduce blood pressure, but the reason why higher concentrations of phenolic acids have more impact on normal rats than hypertensive ones remain to be investigated.

Besides the bioactive substances *Lb. plantarum* can produce in fermented foods, researchers have found that *Lb. plantarum* can also help enhance drug absorption. Febrina A. Saputri et al. [63] reported that providing male New Zealand rabbits with *Lb. plantarum* for 14 days can help enhance the absorption of Amlodipine, which can be used to treat hypertension. The blood concentrations of amlodipine in the *Lb. plantarum IS-10506* group had a higher level compared with those in the control group. This survey suggests that when the animals are supplemented with *Lb. plantarum IS-10506*, their blood flow can be enhanced, which may increase the absorption of amlodipine.

Gut microbiota dysbiosis is another factor that is related to hypertension. According to Yang T. et al., microbial richness and diversity were significantly reduced in the spontaneously hypertensive rats [64]. Additionally, fecal samples of some human hypertension patients were also tested in this research, and a similar dysbiosis pattern was found. The mechanism of this relationship has not been fully clarified. According to Robles-Vera et al., some *lactobacillus* strains can synthesize products such as short-chain fatty acids and lipopolysaccharides [65]. These products can potentially influence host cell physiology. Still, more scientific research is required in this area.

**Table 2:** The ACEI activity of different kinds of foods fermented by *Lb. plantarum*.

*Lactiplantibacillus plantarum*	Fermented Food	Fermentation Condition	ACE Inhibition Effect	References
*Lb. plantarum L69* together with Directed Vat Set starter containing *Lactobacillus bulgaricus* and *Streptococcus thermophilus* (1:1)	skim milk powder	4.5 h, 42 °C, pH = 4.65	*Lb. plantarum L69* contributed to ACEI substances production compared with single Directed Vat Set starter, with ACEI activity reaching 87.14%.	[66]
*Lb. plantarum SPS109*	whey beverage	72 h, 35 °C, pH = 5.5	ACEI activity = 25.70 ± 1.20%	[67]
*Lb. plantarum* previously isolated and identified from Chiapas double cream cheese	reconstituted whole milk.	48 h, 37 °C, pH = 9	59.3 ± 1.6% ACEI activity	[68]
*Lb. plantarum K25* together with yogurt starter *L. delbrueckiissp.* bulgaricus and Streptococcus thermophilus)	yogurt	37 °C, pH = 4.5 ± 0.5, then storing in 4 °C for 21 days	*Lb. plantarum K25* significantly raising the ACEI ratio in contrast to fermentation with the starter only, with the estimation of ACEI activity = 49.3%	[69]
*Lb. plantarum BG 112*	soymilk containing okara flour	32 h	50% ACEI activity	[70]
*Lb. Plantarum* which NCBI Accession is KF806535	soy milk	24 h, 37 °C, 100 rpm	ACEI activity (in-vitro) of peptides all above 70%	[71]
*Lb. plantarum (TISTR 858)*	eggshell membranes	30 °C, 120 rpm	ACE-inhibition corresponding to 49.3% with the concentration of the protein hydrolysates obtained after fermentation up to 2 mg/mL	[72]
*Lb. plantarum 70810*	navy bean milk	2 h, 31 °C	50% inhibiting concentration (IC50) = 109 ± 5.1μg protein/ml	[73]
*Lb. plantarum B1-6*	navy bean milk	3 h, 37 °C	IC50 = 101 ± 2.2 μg protein/mL, in vitro gastrointestinal simulation IC50 = 21 ± 2.1 μg protein/ml	[73]
*Lb. plantarum (NCDO 1193)*	freeze-dried camu-camu powder and soymilk combination	37 °C, 72 h	94.0 ± 1.0% ACEI activity	[74]
*Lb. plantarum 69*	goat milk	35 °C, CaCl_2_ concentration of 0.07%, and Tween-80 concentration of 0.04%	88.91%ACEI activity	[51]
*Lb. plantarum KU15003*	yogurt	fermentation was terminated when the pH reached 4.4 ± 0.1.	IC50 = 0.68 mg/mL	[75]
*Lb. plantarum KU15031 (T3)*	yogurt	fermentation was terminated when the pH reached 4.4 ± 0.1.	IC50 = 0.79 mg/mL	[75]
*Lb. plantarum NK181 (T4)*	yogurt	fermentation was terminated when the pH reached 4.4 ± 0.1.	IC50 = 0.48 mg/mL	[75]
*Lb. plantarum QS670*	milk	37 °C, 48 h	IC50 = 1.26 mg/mL	[57]

### 3.3. Mechanisms of Lipid Lowering by Lb. plantarum

High cholesterol is also one of the risk factors for CVD, which can cause a variety of high mortality diseases such as atherosclerosis and hyperlipidemia. Atherosclerosis is a chronic inflammatory disease, often accompanied by endothelial dysfunction, endometrial lipid deposition, smooth muscle cell proliferation, apoptosis, and necrosis, as well as local and systemic inflammation with the participation of innate and acquired immunity [76]. Hypercholesterolemia is a pathological condition clinically diagnosed with concentrations of total cholesterol (TC), triglyceride (TG), and low-density lipoprotein (LDL) exceeding the standards, along with concentrations of high-density lipoprotein (HDL) being lower than the standards [77]. One of the main causes of these diseases is the accumulation of cholesterol in the arteries. Studies have shown that lowering serum cholesterol concentration by 1% can reduce the incidence of CVD by 2% to 3%, particularly when LDL cholesterol level can be lowered and HDL cholesterol level raised; for patients with atherosclerosis, the low-density lipoprotein cholesterol (LDL-C) goal is <1.8 mmol/L (<70 mg/dL), or a reduction of at least 50% if the baseline level is between 1.8 and 3.5 mmol/L (70 and 135 mg/dL) [78].

Therefore, modern medicine believes that finding drugs that can reduce the lipid levels in the blood and liver is an effective way to prevent CVD. So far, pharmacological treatments such as statins, 3-hydroxy-3-methylglutaryl CoA (HMG CoA) reductase inhibitors, are the most widely used treatments to effectively lower cholesterol, especially LDL-C. However, these drugs are expensive and have harmful side effects, such as liver enzyme abnormalities and rhabdomyolysis [79]. Therefore, scientists hope that a safe substance of natural origin can be found as an alternative to drugs. Probiotics isolated from foods or intestines have great beneficial characteristics, and can be used as functional strains in the treatment and prevention of many diseases. In addition, most lactobacilli have a long history of safe use and are considered Generally Recognized as Safe (GRAS) strains with a high safety profile and low side effects compared to drugs. [80]. As one of them, *Lb. plantarum* has a high cholesterol-lowering capacity in vivo and in vitro, and can significantly improve serum cholesterol levels and reduce the risk of disease [81,82,83,84,85,86,87,88].

The cholesterol-lowering ability of *Lb. plantarum* in vitro has been widely reported, and its possible mechanism has been verified by experiments, including enzymatic deconjugation of bile acids by bile salt hydrolase (BSH), assimilation of cholesterol by probiotics, co-precipitation of cholesterol with deconjugated bile, incorporation of cholesterol into the cellular membranes of probiotics during growth, conversion of cholesterol into coprostanol through cholesterol reductase, and production of short-chain fatty acids upon fermentation [89,90]. However, the mechanism of lipid-lowering in vivo has not been fully clarified. We summarize the possible mechanisms as: (1) regulation of lipid metabolism through signaling pathways; (2) cholesterol reduction through bile acids; (3) regulation of lipid metabolism through intestinal flora; (4) cholesterol reduction through conjugated linoleic acid (CLA) isomerase.

#### 3.3.1. Lowering Cholesterol through Signaling Pathways

Potential probiotic strains of *Lb. plantarum* can regulate lipid metabolism through signal pathways, and the AMPK signaling pathway is one of the classical pathways [91]. *Lb. plantarum* can activate adenosine 5‘-monophosphate (AMP)-activated protein kinase (AMPK), and alter the expression of proteins downstream, thereby regulating the synthesis of cholesterol and fatty acids. In earlier studies, Lee, Choi, et al. [92] found that the way *Lb. plantarum* activates the AMPK pathways was through covalent modification, rather than affecting the expression of AMPK mRNA. By covalent modification, *Lb. plantarum* could increase the phosphorylation levels of AMPK to activate it. Acetyl-CoA carboxylase (ACC) is a receptor protein of AMPK, as well as a key enzyme in fatty acid synthesis, which can be inhibited by the activated AMPK, thus inhibiting lipid synthesis. Besides, activated AMPK signaling promotes fatty acid oxidation by increasing peroxisome proliferation-activated receptor alpha (PPARα) expression, which can avoid the accumulation of fatty acids in the form of TG [93]. Scientists also found that *Lb. plantarum* can significantly down-regulate the expression of lipogenic proteins in liver tissue, such as sterol regulatory element-binding protein 1c (SREBP-1c) and stearoyl-CoA desaturase 1 (SCD-1) [94]. All of this suggests that *Lb. plantarum* can regulate liver lipid metabolism to some extent through the AMPK pathway, increasing fatty acid oxidation while decreasing lipid biosynthesis.

This mechanism has been confirmed in many species of *Lb. plantarum*. Yue Teng et al. [95] conducted experiments using *Lb. plantarum LP104* isolated from kimchi. They randomly divided the mice into the normal-fat diet (NFD) group, a high-fat diet (HFD) group, and HFD + LP104 group. They found that in the HFD + *Lb. plantarum* 104 group, the phosphorylation level of AMPK was increased, and the level of ACC was reduced compared to the HFD group. Furthermore, the expression of lipogenic proteins SREBP-1c and SCD-1 was down-regulated, while the expression of PPARα was significantly increased. Meanwhile, experimental data showed that supplementation with *Lb. plantarum LP104* significantly reduced the elevated TC, TG, and LDL levels caused by the high-fat diet. The same results have been found in *Lb. plantarum NCU116* [96], *Lb. plantarum NA136* [97], and *Lb. plantarum S58* [94].

#### 3.3.2. Lowering Cholesterol through Bile Acids

*Lb. plantarum* can also lower serum cholesterol levels through bile acids. Bile acid is one of the components of bile, and in alkaline bile, it is often present as a potassium salt or sodium salt, hence the name bile salt. Bile salt is closely related to the balance of cholesterol in the body. Clinical studies have shown that bile acid levels are significantly higher in patients with hyperlipidemia, non-alcoholic steatohepatitis, and fatty liver than in normal people [98], and since cholesterol is a precursor to the de novo synthesis of bile salts [99], promoting the catabolism of bile salts may be an effective way to lower cholesterol.

A 2009 study by Sridevi and Prabhune [100] demonstrated that many *Lb. plantarum* have high bile salt hydrolase (BSH) activity, which can regulate bile acid metabolism and lower cholesterol levels. BSH is an enzyme of high biological significance belonging to the family chologlycine hydrolase (EC 3.5.1.1 1) that catalyzes the conversion of conjugated bile salts into free bile salts and amino acids. Conjugated bile salts are very soluble, and most of them can be reabsorbed through enterohepatic circulation. In contrast, unconjugated bile salts or free bile salts are insoluble and are reabsorbed less efficiently, so they will be excreted with feces [101]. After BSH catalysis, the bile acid content in the body decreases, and more cholesterol is needed if bile salts are to be formed. With the high activity of BSH, *Lb. plantarum* can increase bile acid consumption and promote the de novo synthesis of cholesterol into bile acids, ultimately reducing the body’s cholesterol levels.

This process is adjusted by the expression of a variety of proteins, including farnesoid X receptor (FXR), liver X receptor (LXR), and cholesterol 7α-hydroxylase (CYP7A1). Wang Huang et al. [102] fed mice with Lb. plantarum AR113, which has high BSH activity. They found that the serum TC and LDL-C levels decreased significantly, and the serum HDL-C level was increased in the *Lb. plantarum AR113* group compared with mice in the control groups. Meanwhile, the mRNA levels of FXR were significantly down-regulated, and mRNA expression of CYP7A1 and LXR were significantly upregulated in the AR113 group, which means that *Lb. plantarum AR113* can regulate the metabolism of bile acids to affect cholesterol content. Other studies have obtained similar findings in other *Lb. plantarum* with high BSH activity [103,104].

In addition to regulating bile acid metabolism through high BSH activity, *Lb. plantarum* can decrease serum cholesterol levels by promoting the combination of free bile salt and cholesterol to form precipitation, which is hard to absorb. Aarti [105] isolated several strains from breast-fed infants’ feces and cultured them in MRS broth to which sodium glycinamide and sodium taurocholate were added. The content of bile acids released from the conjugated bile salts sodium glycinamide and sodium taurocholate were then utilized as criteria to determine the ability of different strains to remove cholesterol. The method described by Walker and Gilliland was used to detect the cholic acid level and colorimetric analyses to detect cholesterol level. The experimental results proved that among all these strains, *Lb. plantarum GD2* had the best capacity to precipitate cholesterol, while it could survive at low PH and high bile concentration, which indicates that *Lb. plantarum GD2* has a good ability for cholesterol scavenging. The exact mechanism still needs to be determined by further studies.

**Figure 3:**
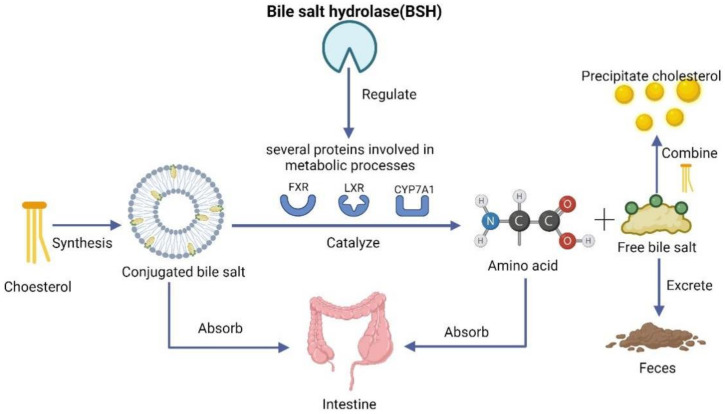
**Mechanism of cholesterol lowering by *Lactiplantibacillus plantarum* via bile acids.***Lactiplantibacillus* plantarum reduces cholesterol by regulating the metabolism of bile acids that consume cholesterol, as well as promoting the combination of bile acids and cholesterol into precipitates. (FXR—farnesoid X receptor, LXR—liver X receptor, CYP7A1—cholesterol 7α-hydroxylase) (Created with Biorender.com).

The two ways in which *Lb. plantarum* regulates bile acid levels and lowers cholesterol are shown in Figure 3. These findings indicate that *Lb. plantarum* can regulate bile acid metabolism through BSH, and promote the combination of free bile salts and cholesterol to reduce lipids. Therefore, cholesterol-lowering via bile acids may be a promising approach for the use of *Lb. plantarum* in the treatment of CVD.

#### 3.3.3. Lowering Cholesterol through Intestinal Flora

Accumulating evidence suggests that gut dysbiosis induced by a high-fat diet promotes the development of hypercholesterolemia, obesity, and other metabolic syndromes. Recent studies have highlighted the importance of the gastrointestinal microbiome in regulating host health and disease [106]. Scientists have suggested that *Lb. plantarum* could increase the species richness and biodiversity of intestinal flora, as evidenced by an increase in beneficial microorganisms and a decrease in pathogenic bacteria. Shao, Y., Huo, D., Peng, Q., et al. [107] applied a metagenomic approach to indicate that the consumption of probiotics can regulate the intestinal microbial structure in hyperlipidaemic patients. Their research showed that the genera *Bifidobacterium*, *Lactobacillus*, *Akkermansia*, and *Faecalibacterium* were significantly increased in the groups fed with *Lb. plantarum*, while the genera *Clostridium*, *Natranaerovirga*, *Fervidicella*, *Roseburia*, *Gemella*, *Escherichia/Shigella*, and *Odoribacter* were significantly increased in the hyperlipidemic group. The intrinsic mechanism of lipid-lowering by intestinal flora has not been fully clarified, although it is a current research hotspot. We look forward to the latest research results in the future.

#### 3.3.4. Lowering Cholesterol through Conjugated Linoleic Acid (CLA) Isomerase

In addition to the mechanism above, *Lb. plantarum* can produce conjugated linoleic acid (CLA) isomerase to synthetic CLA, thus reducing cholesterol and treating hyperlipidaemia.

CLA is not a single kind of linoleic acid but a series of linoleic acid isomers, whose functions are caused by the coordinated regulation of metabolic pathways by multiple CLA isomers, such as cis-9, trans-11, trans-10, and cis-12, playing an important role in fat uptake and lipid metabolism. However, the requirements for the chemical synthesis of linoleic acid are complex and there are many by-products. Thus, the biosynthesis of linoleic acid isomerase through microorganisms is believed to be a great alternative approach. Some bacteria, especially *Lb. plantarum ZS2058*, screened from Chinese traditional food pickles, had high linoleate isomerase activity, and has proved to be the most efficient strain of *lactobacillus*. Through localization and purification of linoleic acid isomerase, it was determined that CLA isomerase catalyzed hydrated reaction in microbial cells to form 10- HOE, which is the intermediate of CLA synthesis, and after multi-step reaction generated the CLA with biological activity [108].

The same transformation mechanism exists in *Lb. plantarum AKU1009A* [109]. In addition, a study of *L. plantarum CGMCC8198* showed that it had a homologous sequence with the isomerase gene of *L. plantarum ZS2058*, and linoleic acid isomer could be produced by fermentation with Acer Truncatum Bunge seeds oil. This has laid a certain foundation for microbial production of *Lb. plantarum* [110]. In the experiment of Chen L. et al. [111], dietary supplementation of conjugated CLA can increase the abundance of lipid metabolism-related bacteria and significantly reduce fat deposition in mice, further evidence of its cholesterol-lowering effect.

### 3.4. Mechanisms of Glucose Lowering by Lb. plantarum

The concentration of glucose in the blood is called the serum glucose level. A normal serum glucose level plays an important role in maintaining the human body’s metabolism and the basic functioning of all organs. Thus, if the blood glucose level is too high or too low, it can cause damage to people’s health. Hyperglycemia can increase the risk of diabetes, lead to poor immunity, and cause CVD. Meanwhile, if glucose is not utilized by the body in a timely manner, it may lead to low energy supply, weariness, and depression.

*Lb. plantarum* can regulate the serum glucose level and inhibit diabetes [112]. Ahtesham Hussain [113] used *Lb. plantarum LB818* to feed mice and found that high-fat diet (HFD) mice had a lower serum glucose level when treated with *Lb. plantarum LB818*. Taking the changes in TC, TG, HDL, alanine aminotransferase (ALT), and aspartate aminotransferase (AST) into account, it can be concluded that *Lb. plantarum LB818* can display anti-diabetic effects by preventing weight gain, lowering fat, and balancing glucose levels. Similar results were obtained in a study conducted by U. Andersson et al. [114]. They found that feeding with *Lb. plantarum DSM 15313* can enhance the glucose elimination and insulin response of mice. Moreover, in another report, rats showed a decrease in serum glucose levels after 28 days of taking *Lb. plantarum 49* and *Lb. plantarum 201* [115].

**Figure 4:**
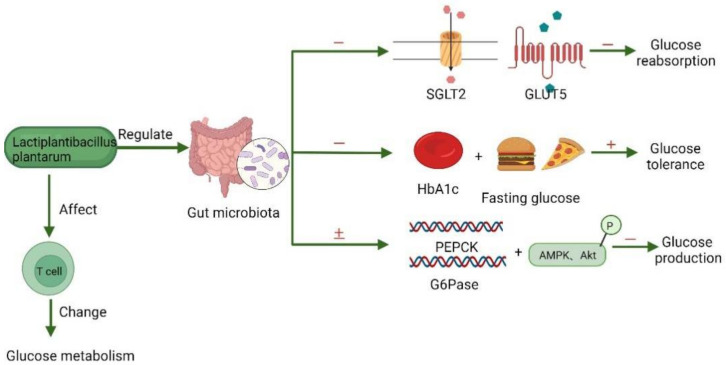
**The blood glucose lowering mechanism of *Lactiplantibacillus plantarum.****Lactiplantibacillus plantarum* lowering glucose level through gut microbiota, which can regulate the expression of Phosphoenolpyruvate carboxykinase (PEPCK) and Glucose-6-phosphatase (G6Pase), as well the Adenosine 5‘-monophosphate activated protein kinase (AMPK)/protein kinase B (Akt) signaling pathway, meanwhile reducing the level of transporter SGLT2, GLUT5, glycated hemoglobin (HbA1c), and fasting glucose. Immune cell function is regarded as a possible mechanism but still needs further study. (“+” means upregulation, “−“ means downregulation and “±“ means upregulation or downregulation) (Created with Biorender.com).

Numerous related experiments were conducted to validate the hypoglycemic effect of *Lb. plantarum* and explore the underlying mechanism of *Lb. plantarum*. Although the glucose-lowering mechanism remained to be elucidated, the most likely explanation was related to gut microbiota. Sen Lin [116] detected the changes in glucose concentrations of piglets that were fed with *Lb. plantarum* and analyzed the microbiota profile in fecal samples. Based on the results, they speculated that the way *Lb. plantarum* affected glucose homeostasis may be associated with modulating the relative abundances of gut microbial genera. *Lb. plantarum* can alter the host’s gut microbiota, leading to the upregulation of specific glucose, which means a decrease in glucose reabsorption and a decrease in fasting glucose and glycated hemoglobin (HbA1c) levels. Moreover, with the higher glucose tolerance, the protein kinase B (Akt) and AMPK phosphorylation, as well as the mRNA expression levels of Phosphoenolpyruvate carboxykinase (PEPCK) and Glu-cose-6-phosphatase (G6Pase) are altered, resulting in the decrease of glucose production. The possible mechanism by which gut microbiota affect glucose metabolism is shown in Figure 4.

In addition, studies have shown that *Lb. plantarum* can regulate signal pathways through intestinal flora [117,118,119]. It is reported that mice fed with *Lb. plantarum HAC01* had a lower level of fasting glucose and glycated hemoglobin (HbA1c) level and performed better in the oral glucose tolerance test (OGTT), which suggests the anti-hyperglycemic effect of the strain, and implies that it can improve glucose intolerance. Through the study of the expression of genes and proteins related to glucose metabolism in the liver, scientists found that it was able to increase Akt and AMPK phosphorylation, and decrease PEPCK andG6Pase mRNA expression levels in the liver, therefore affecting endogenous glucose production in the liver [120]. Some researchers have also proved that *Lb. plantarum* could improve the utilization of glucose. Rats fed with a high-fructose diet exhibited upregulation of specific glucose transporter SGLT2 and specific fructose transporter GLUT5, which may lead to the higher fructose level in the blood and kidney of rats. A supplement of *Lb. plantarum* may restrict the reabsorption of glucose [121]. Apart from utilizing glucose, *Lb. plantarum* can also adapt to glucose-limited conditions. Studies of the genotypic and proteomic changes in *Lb. plantarum P-8* in response to long-term (3 years) glucose limitation, explored various mechanisms for survival, which consist of altering the cell envelope, activating the phosphotransferase system, accumulating and consuming amino acids, reducing glucose intake, and increasing the generation of glucose or ATP in response to glucose starvation [122].

Aside from that, *Lb. plantarum OLL2712* could regulate glucose metabolism without the reduction of body weight, and this effect may be connected with the functioning of immune cells, such as T cells. However, whether immune cells can change glucose metabolism requires further study [123].

## 4. Recent Developments in *Lb. plantarum*-Fermented Food

**Figure 5:**
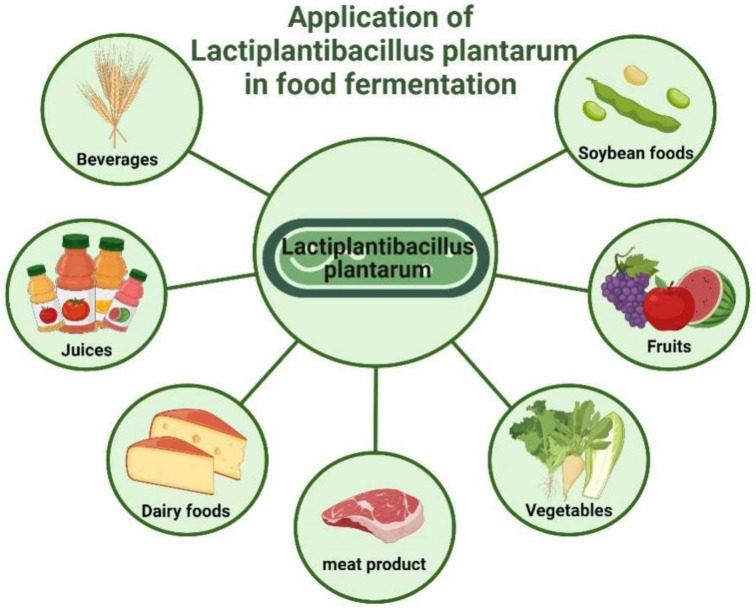
**The application of *Lactiplantibacillus plantarum* in several different types of food.***Lactiplantibacillus plantarum* has been widely used as a fermenting strain in the production of a wide range of fermented foods. (Created with Biorender.com).

As mentioned above, *Lb. plantarum* has the potential to be developed as a probiotic because of its excellent anti-cardiovascular properties, as well as its good tolerance in the gastrointestinal tract, which is important for human health. In addition, some studies have demonstrated that foods fermented with it have better health benefits than before fermentation, and may have the ability to be developed into probiotic functional foods. The applications of *Lb. plantarum* in different types of foods, and the corresponding effects of its fermented foods, are shown in Figure 5 and Table 3.

### 4.1. Fermented Food with Antioxidant Function

Fruits and vegetables are rich in phenolic substances, carotenoids, flavonoids, and vitamin C, which have good antioxidant effects. Therefore, scientists hope to develop functional beverages based on fruit and vegetable juices and improve their antioxidant capacity through fermentation. In earlier research, some fermented juices with antioxidant functions, such as apple, orange, jujube, and coconut juices have been produced and proven to have a better ability to scavenge free radicals. In recent years, there have also been many reports on the antioxidant functional juice fermented by *Lb. plantarum*. Wu, Li, et al. [143] compared the antioxidant activity of blueberry and blackberry juices before and after fermentation based on ABTS methods, and they found that the ABTS radical scavenging activity of blackberry and blueberry juices fermented with Lactobacillus plantarum increased by 53.3% and 64.0%, respectively, compared to the juice before fermentation. The same results were also reported in fermented vegetable juice. Zhang, Duan, et al. [144] also found that the DPPH radical scavenging rate and ABTS free radical scavenging rate of carrot juice after 72 h of fermentation by *Lb. plantarum WZ-01* were increased to some extent compared to their pre-fermentation state. These results suggest that fermentation by *Lb. plantarum* can effectively enhance the free radical scavenging activity of juices, and fermented fruit and vegetable juices can be developed as a good antioxidant functional beverage.

In addition to fruit and vegetable juice, there are also reports on the application of *Lb. plantarum* in the fermentation of other types of foods, including dairy products [132], soybean products [129], fermented sausages [139]. All these types of fermented foods show better antioxidant capacity in vitro compared with prior to fermentation.

### 4.2. Fermented Food with Cholesterol-Lowering Function

The development of *Lb. plantarum*-fermented foods with cholesterol-lowering effects have achieved some results, including dairy products, soybean products, fruits and vegetables, aquatic products. As early as 2015, Jeon, Lee, et al. [145] isolated a strain of lactic acid bacteria from kimchi that could lower cholesterol in vitro, named *Lb. plantarum EM*. Four years later, they utilized *Lb. plantarum EM* as a fermenting strain in cabbage-apple juice to investigate the health-promoting effects of fermented juice on high-cholesterol diet rats. It was found that the serum levels of TG, TC, and LDL-C were greatly reduced, and HDL-C levels were increased in rats fed with fermented juice compared to unfermented juice. Many scientists have obtained similar results with different types of fermented foods. Cao, Wu, et al. [131] pointed out that soy extract fermented by *Lb. plantarum* could regulate lipid metabolism through signaling pathways and thus lower cholesterol. Li, Wu, et al. [135] analyzed the microbial diversity of rat feces and found that skim milk fermented with *Lb. plantarum WW* could regulate intestinal flora and lower cholesterol levels. Hu, Zheng, et al. [128] found that *Lb. plantarum FZU3013*-fermented Laminaria japonica could affect the expression of genes involved in lipid metabolism and bile acid homeostasis in rats.

### 4.3. Fermented Food with Blood Pressure Lowering Function

There are few reports on the hypotensive effects of *Lb. plantarum*-fermented foods, and most of those reports focused on fermented foods that can inhibit ACE activity and are mainly fermented dairy products. Some research results are shown in Table 2 above. in addition to dairy products, the same findings have been reported for other fermented foods, such as soy milk [71], fermented sausages [146], and fruits like guava [147]. However, due to the lack of relevant studies, it cannot be ascertained whether fermented foods are effective in in vivo experiments.

### 4.4. Fermented Food with Hypoglycemic Function

Several studies have confirmed the hypoglycemic effect of *Lb. plantarum*-fermented foods. Zhong, Abdullah, et al. [126] used blueberry as a fermentation substrate. Blueberries are widely considered to have a good antidiabetic ability and the potential to treat early-stage diabetes. In their research, they found that, compared with the non-fermented blueberry juice, the fumarate contents in blueberry juice fermented by *Lb. plantarum* were significantly increased, which could maintain glucose homeostasis. In addition, fermentation altered the concentrations of phenolic compounds in blueberry juice, which promoted glucose consumption. They employed HepG2 cell lines as a model to confirm that fermented juice significantly enhanced cellular glucose consumption [148]. In addition to this, *Lb. plantarum* fermentation also increases α-Amylase and α-glucosidase inhibitory activity, thus reducing carbohydrate hydrolysis for the effective control of diabetes [149].

### 4.5. Other Applications of Lb. plantarum

In conclusion, it can be seen that *Lb. plantarum*-fermented foods show good effects in in vitro experiments, and have the potential to be developed into probiotic products. Moreover, in addition to conferring additional functional characteristics to the foods, *Lb. plantarum* fermentation may improve the properties of traditional fermented foods. Compared with unfermented food, fermented food can produce bacteriocins with good antimicrobial properties and extended storage time [150]; it may also have better stability [151], and its flavor will be improved to meet consumers’ demands [124]. At present, there are various kinds of functional fermented foods reported covering the basic diet, such as dairy products, fruits, vegetables, meat products, soybeans, and cereals. These factors comprehensively show that developing *Lb. plantarum*-fermented foods and supplementing these foods in daily life may be of great significance to human health.

## 5. Conclusions

In the context of high CVD incidence worldwide, in addition to common pharmacological treatments, probiotics and probiotic functional foods have become important means of preventing and alleviating such diseases. *Lb. plantarum* has great potential in the prevention and treatment of CVD and could be developed as a probiotic to play a healthy role in the human body. Its mechanisms include increasing antioxidant levels and maintaining the balance of blood glucose, blood lipids, and blood pressure. At the same time, studies have found that *Lb. plantarum* has a wide range of applications in fermented foods such as beverages and cheese. A variety of fermented foods have the effect of preventing and alleviating CVD. Research and optimization of the composition, technology, properties, and other related characteristics of *Lb. plantarum*-fermented foods can assist scientists in improving the quality of these foods and their probiotic activity, ultimately alleviating patients’ CVD symptoms. Therefore, *Lb. plantarum* functional fermented foods have broad application prospects in reducing the risk of CVD and its treatment. In the future, we can reduce the risk of CVD and alleviate CVD by supplementing *Lb. plantarum* fermented foods to our daily diet. However, the therapeutic effects of *Lb. plantarum*-fermented food on CVD have not been clinically verified, and the function of *Lb. plantarum* in actual production is affected by temperature, pH value, food composition, and other food microorganisms. Therefore, in order to develop functional foods better, it is necessary to explore the optimal production conditions further.

## Figures and Tables

**Table 3:** The effects of *Lb. plantarum* fermented foods in the prevention of different diseases.

Types of Food	Fermented Food	*Lb. plantarum*	Function	Mechanism	References
Fruit and vegetable juice	charantia juice	*Lb. plantarum NCU116*	antioxidant	increase the content of phenolic compounds and promote the biotransformation to provide stronger antioxidant properties	[124]
	papaya juice	*Lb. plantarum GIM1.140*	antioxidant	increase the content of total flavonoids and improve inhibition of DPPH free radicals	[125]
	blueberry juice	A variety of mixed strains including *Lb. plantarum*	anti-diabetes	maintain glucose homeostasis and promote glucose consumption	[126]
	green loofah	*Lb. plantarum SU4*	cholesterol lowering	high bile acid lowering capacity in vitro and in vivo to promote cholesterol consumption	[127]
Aquatic product	laminaria japonica	*Lb. plantarum FZU3013*	cholesterol lowering	reduce expression levels of genes involved in lipid metabolism and bile acid homeostasis to promote cholesterol consumption	[128]
Soybean products	black Soymilk	*Lb. plantarum BCRC 10357*	antioxidant	increase the ferric reducing antioxidant capacity	[129]
	soy milk added with cuminum cyminum essential oil	*Lb. plantarum* *A7 (KC 355240)*	anti-diabetes and cholesterol lowering	significantly reduces postprandial serum glucose concentrations and TG levels.	[130]
	soy extract	*Lb. plantarum WW*	cholesterol lowering	regulate the expression levels of genes involved in lipid metabolism and oxidation-reduction processes to promote cholesterol catabolism	[131]
Dairy products	orange juice-milk based beverage	*Lb. plantarum (TR-7, TR-71, TR-14)*	antioxidant	increase the content of carotenoids and the total antioxidant activity	[132]
	kalari cheese	*Lb. plantarum NCDC 012*	anti-diabetes	produce a variety of bioactive peptides to enhance the inhibitory activity of α-amylase and α-glucosidase, and inhibit carbohydrate decomposition to lower glucose	[133]
	goat milk	*Lb. plantarum C4*	blood pressure lowering	enhance the ACE inhibitory activity by fermentation	[134]
	skim milk	*Lb. plantarum WW*	cholesterol lowering	regulate the intestinal flora and lower cholesterol levels	[135]
	cheese	*Lb. plantarum VC213*	cholesterol lowering	significantly lower cholesterol content than before fermentation	[136]
Cereal grains	rice bran and wheat bran	*Lb. plantarum NCU116*	antioxidant	enhance the hydroxyl radical-scavenging activity and the oxygen radical-quenching activity	[137]
	whole-grain oats	*Lb. plantarum B1-6*	blood pressure lowering	present higher ACE inhibitory activities	[138]
Meat products	Chinese fermented sausages	*Lb. plantarum CD101*	antioxidant	reduce pH, and promote the formation of antioxidant peptides	[139]
	fermented meat patty	*Lb. plantarum PTCC 1745*	antioxidant	radical scavenging activity significantly higher than before fermentation	[140]
	fermented camel sausages	*Lb. plantarum KX881772*	anti-diabetes	higher α-amylase and higher α-glucosidase inhibitions to control diabetes by reducing carbohydrate hydrolysis	[141]
	fermented sausage	*Lb. plantarum CD101*	blood pressure lowering	significantly increase the ACE inhibitory activity	[142]

## Data Availability

Not applicable.
